# Basic amino acid-mediated cationic amphiphilic surfaces for antimicrobial pH monitoring sensor with wound healing effects

**DOI:** 10.1186/s40824-023-00355-0

**Published:** 2023-02-17

**Authors:** Dong Uk Lee, Se-Chang Kim, Dong Yun Choi, Won-Kyo Jung, Myung Jun Moon

**Affiliations:** 1 Biomedical Manufacturing Technology Center, Korea Institute of Industrial Technology, Yeongcheon, 38822 Republic of Korea; 2 Department of Industrial Chemistry, Pukyong National University, Busan, 48513 Republic of Korea; 3 Major of Biomedical Engineering, Division of Smart Healthcare, College of Information Technology and Convergence and New-Senior Healthcare Innovation Center (BK21 Plus), Pukyong National University, Busan, 48513 Korea; 4 Marine Integrated Biomedical Technology Center, The National Key Research Institutes in Universities, Pukyong National University, Busan, 48513 Korea; 5 Research Center for Marine Integrated Bionics Technology, Pukyong National University, Busan, 48513 Republic of Korea

**Keywords:** Antimicrobial, Biofilm inhibition, pH sensor, Wearable biomedical device, Wound healing

## Abstract

**Background:**

The wound healing process is a complex cascade of physiological events, which are vulnerable to both our body status and external factors and whose impairment could lead to chronic wounds or wound healing impediments. Conventional wound healing materials are widely used in clinical management, however, they do not usually prevent wounds from being infected by bacteria or viruses. Therefore, simultaneous wound status monitoring and prevention of microbial infection are required to promote healing in clinical wound management.

**Methods:**

Basic amino acid-modified surfaces were fabricated in a water-based process via a peptide coupling reaction. Specimens were analyzed and characterized by X-ray photoelectron spectroscopy, Kelvin probe force microscopy, atomic force microscopy, contact angle, and molecular electrostatic potential via Gaussian 09. Antimicrobial and biofilm inhibition tests were conducted on *Escherichia coli* and *Staphylococcus epidermidis*. Biocompatibility was determined through cytotoxicity tests on human epithelial keratinocytes and human dermal fibroblasts. Wound healing efficacy was confirmed by mouse wound healing and cell staining tests. Workability of the pH sensor on basic amino acid-modified surfaces was evaluated on normal human skin and *Staphylococcus epidermidis* suspension, and in vivo conditions.

**Results:**

Basic amino acids (lysine and arginine) have pH-dependent zwitterionic functional groups. The basic amino acid-modified surfaces had antifouling and antimicrobial properties similar to those of cationic antimicrobial peptides because zwitterionic functional groups have intrinsic cationic amphiphilic characteristics. Compared with untreated polyimide and modified anionic acid (leucine), basic amino acid-modified polyimide surfaces displayed excellent bactericidal, antifouling (reduction ~ 99.6%) and biofilm inhibition performance. The basic amino acid-modified polyimide surfaces also exhibited wound healing efficacy and excellent biocompatibility, confirmed by cytotoxicity and ICR mouse wound healing tests. The basic amino acid-modified surface-based pH monitoring sensor was workable (sensitivity 20 mV pH^−1^) under various pH and bacterial contamination conditions.

**Conclusion:**

Here, we developed a biocompatible and pH-monitorable wound healing dressing with antimicrobial activity via basic amino acid-mediated surface modification, creating cationic amphiphilic surfaces. Basic amino acid-modified polyimide is promising for monitoring wounds, protecting them from microbial infection, and promoting their healing. Our findings are expected to contribute to wound management and could be expanded to various wearable healthcare devices for clinical, biomedical, and healthcare applications.

**Supplementary Information:**

The online version contains supplementary material available at 10.1186/s40824-023-00355-0.

## Introduction

Potentiometric pH monitoring sensors are one of the wearable biomedical devices that monitor wound status on skin [1–3]. They offer several advantages in real-time health monitoring, such as miniaturization, simple hardware requirements, low cost, and reusability [4, 5]. The pH sensor uses biochemical markers (pH) that monitor the physiological response by analyzing human perspiration and providing health status-related information such as vascularization and inflammation during wound healing [6, 7]. The pH of normal healthy human skin is in the range of 4–6.5 [2, 8], however, cystic fibrosis and wound infection might serve as the pH slightly alkaline (> 6.5) because of the defective bicarbonate-resorption and the presence of bacteria, respectively [9–11]. Therefore, the detection of pH changes is effective way for real-time wound status monitoring.

However, bacterial infection associated with wearable biomedical devices has become a global healthcare challenge as it impairs wound healing process and could cause the wounds to become chronic [12, 13]. Moreover, the entry of microbial pathogens in wounds exacerbates pain associated with the injury/lesion and unnecessarily increases health care and socioeconomic costs [14–16]. Therefore, the next generation of wound healing dressings should be designed to prevent and promptly detect microbial infection during wound treatment. Smart sensor devices that measure wound healing status in real-time will facilitate the appropriate clinical treatment decisions, prevent bacterial infection, promote wound healing, and manage chronic wound progression.

Wound healing dressings that have various forms in film, sponge, hydrogel and others are currently the main choices in clinical wound management [13]. However, it is difficult to simultaneously obtain real-time wound status information while controlling infection under the passive treatment provided by current wound healing dressings [17]. Therefore, pH-monitorable wound-healing active sensors have attracted attention as novel medical devices for wound management. The pH-monitorable wound healing dressings with various hydrogel coatings have been studied extensively [13, 18]. As a wound healing sensor device, however, the hydrogel layer must have the following properties: (i) low impedance for measuring potentiometric pH values; (ii) flexibility, swellability, and other physical properties essential application to human skin wounds; and (iii) antimicrobial activity to prevent wound infection during use.

Polyimide (PI) is an intrinsic bio-friendly material widely used in various industries [19, 20] because it has biocompatibility, physical strength, electrical insulation, thermal stability, and other desirable characteristics [21, 22]. Thus, PI could also be suitable as a substrate for skin-contacting sensor devices and other various sensor applications. However, the PI surface is unsuitable for wound healing as it has poor hemocompatibility and low oxygen permeability [23–25]. Hence, PI should be subjected to further treatment or functional layer modification to render it feasible for use in wound treatment sensors.

**Scheme 1:**
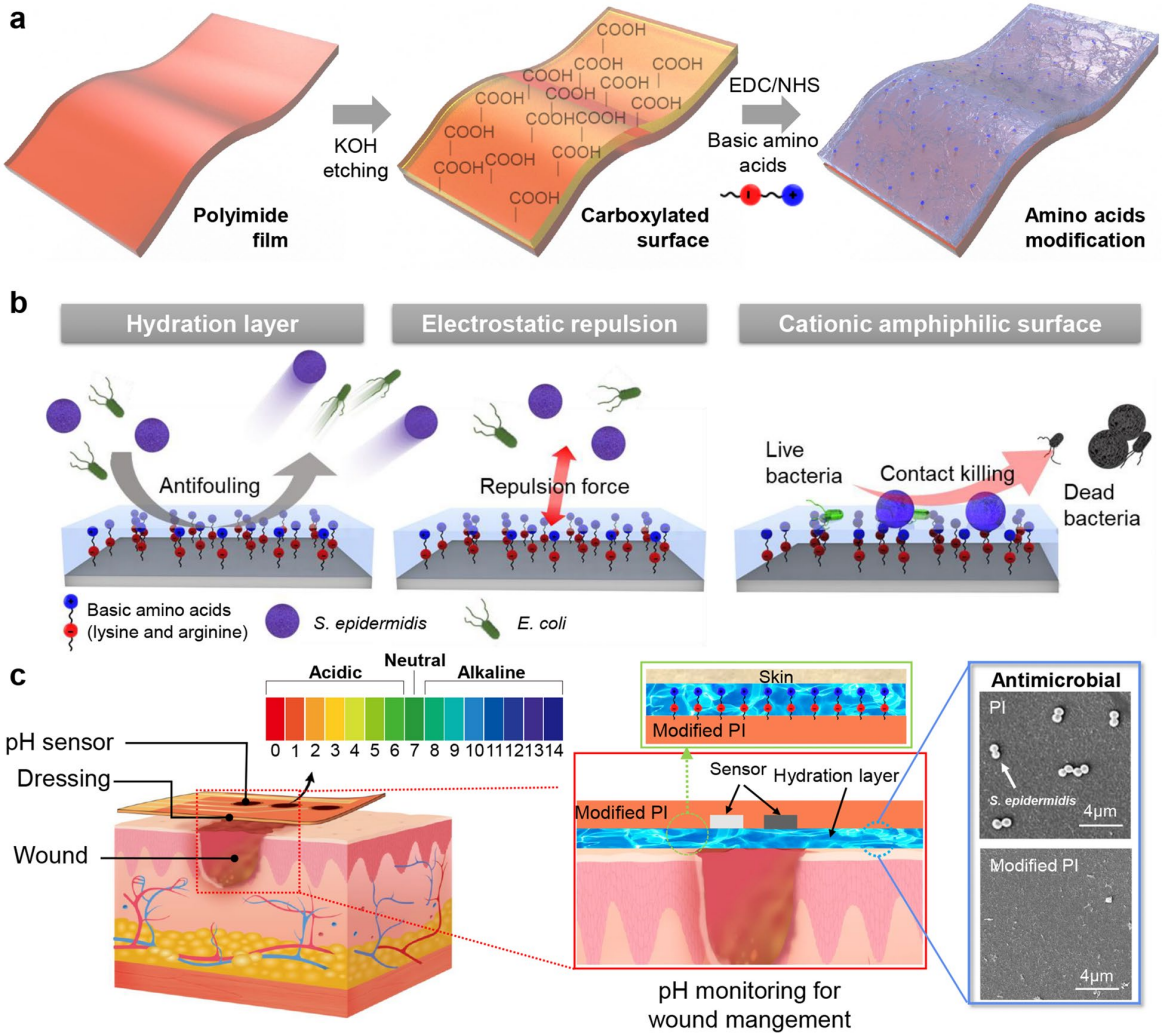
Schematic of preparation, mechanisms, and design of pH-monitorable wound healing dressing with antimicrobial activity. (**a**) Amino acid-mediated surface modification process. (**b**) Mechanism of antimicrobial activity of basic amino acid-modified surfaces. (**c**) Schematic illustration of design of pH-monitorable wound healing dressing devices comprising basic amino-acid-modified polyimide (PI) film

The PI surface can be modified with amino acids via a simple waterborne dipping process (Scheme 1a and Fig. S1) and does not affect physical properties, as reported previously [26]. The basic amino acids (lysine (Lys) and arginine (Arg)) have pH-dependent zwitterionic properties and serve as zwitterionic functional groups on amino acid-modified surfaces at pH 4–8, such as biomedical conditions (Fig. S2). The zwitterionic functionalized surface has mainly two antifouling mechanisms; it forms a hydration layer on a surface that inhibits bacterial contamination by steric repulsion and hinders bacterial adhesion by electrostatic repulsion force. Furthermore, a surface modified with basic amino acids could kill the bacteria because it has cationic amphiphilic properties (Scheme 1b).

In the present study, we introduced the development of a pH-monitorable wound healing dressing film comprising basic amino acid-modified PI and suitable for pH monitoring, wound healing dressing, and the prevention of infection (Scheme 1c). The developed basic amino-acid modification-mediated pH-monitorable wound healing dressing showed antimicrobial activities (reduced bacterial adhesion ~ 99.66% against *Escherichia coli* and 99.63% against *Staphylococcus epidermidis*) and excellent antibiofilm performance. Furthermore, it can be helpful in wound healing due to the hydration layer by means of zwitterionic hygroscopic properties [27, 28] and monitor the wound condition and microbial pathogen infections. The present study shows that pH-monitorable wound healing dressings composed of basic amino acid-modified PI are considered promising as wearable biomedical devices for clinical, biomedical and healthcare applications.

## Methods

### Surface modification with amino acids

Surface pretreatment and modification were performed according to a previously reported method [26]. The polyimide (PI) film was immersed in 1 M potassium hydroxide (KOH) (Duksan, Ansan, Republic of Korea) at 60 °C for 15 min. The film was then washed with deionized (DI) water and the residual ions were removed by immersion in DI water for 10 min. The KOH-treated film was immersed in 0.2 M hydrochloric acid (HCl; Duksan) and immediately washed with DI water.

A 0.1 M 2-(*N*-morpholino)ethanesulfonic acid (MES; Thermo Fisher Scientific, Waltham, MA, USA) buffer solution was prepared by dissolving MES in water and adjusting the pH to 5.5 with 1 M KOH. Then 1.92 g of 1-ethyl-3-(3-dimethylaminopropyl)carbodiimide (EDC; TCI, Tokyo, Japan) and 0.46 g *N*-hydroxysuccinimide (NHS; TCI) were dissolved in 200 mL MES buffer solution (pH 5.5). The carboxylated PI film was surface-modified by immersion in EDC/NHS in MES buffer solution at 0 °C for 15 min. Amino acids (Leu, Lys, and Arg); Sigma-Aldrich, St. Louis, MO, USA) were dissolved in sodium carbonate-sodium bicarbonate Na_2_CO_3_-NaHCO_3_ (Duksan) buffer solution in a separate beaker to prepare three 0.1 M amino acid solutions at pH 8.5. The PI film was then modified by immersion in the amino acid solutions for 12 h.

### Characterization of the surface properties

ATR-FTIR spectroscopy (iS10; with ZnSe crystal kit, Thermo Fisher Scientific) was used to characterize the surface modifications of the PI film. Each specimen was washed with ethanol (Duksan), dried with N_2_ gas, and analyzed over 32 scans at 4 cm^−1^ resolution to generate spectra in the 650–4,000 cm^−1^ range. X-ray photoelectron spectroscopy (XPS) was used to confirm that the film surface was effectively modified with the amino acids. XPS (Multilab 2000; Thermo Fisher Scientific) analyzed the specimens in the 287–1,200 eV range and at 90° angle using an Al K-alpha source (225 W) and monochromatic mode. To compare the water layer formation on the surface based on the surface hydrophilicity, each PI film (10 cm × 10 cm) was immersed in DI water for 5 min; the moisture on the surface was removed, and the weight was measured. This procedure was performed at least five times for all specimens and the results were compared.

### Bacterial cultivation

Gram-negative *Escherichia coli* (*E. coli;* ATCC 11775) were dispersed in 20 g L^−1^ Luria Bertani broth (LB; Sigma Aldrich) and incubated in the dark at 37 °C for 24 h. Gram-positive *Staphylococcus epidermidis* (*S. epidermidis;* ATCC 12228) were dispersed in 30 g L^−1^ tryptic soy broth (TSB; Thermo Fisher Scientific) and incubated at 37 °C in the dark for 24 h. All bacteria were centrifuged at 600 × *g* and 5 °C for 5 min. The supernatants were discarded and the bacterial precipitates were suspended in phosphate-buffered saline (PBS; Thermo Fisher Scientific). The bacterial precipitates were washed twice by centrifugation with PBS. The bacterial suspensions were dispersed in PBS, stored at 2 °C, and used within 12 h. The *E. coli* and *S. epidermidis* densities in PBS suspension were 4 × 10^7^ CFU mL^−1^ and 2 × 10^7^ CFU mL^−1^, respectively.

### Evaluation of antimicrobial activities

The bacterial evaluation method used was previously reported [26]. Ten grams LB broth or TSB and 20 g agar were dissolved in 1 L DI water in an autoclavable bottle by stirring at 90 °C and the solution was autoclaved in a convection oven at 140 °C for 30 min. The agar solution was then stored at 20 °C, cooled to 50 °C, and poured into Petri dishes. The agar plates were then refrigerated for 1 h. Each 2 cm × 2 cm PI film was placed in a 50 mL conical tube and 20 mL bacterial suspension was poured onto it. The conical tubes containing the PI films were stored in a water bath in the dark at 37 °C for 24 h.

To quantify the bacteria adhering to the surfaces, the PI films were gently washed twice with PBS solution and vortexed for 30 s to detach the unattached bacteria. The films were then vortexed for 2 min with 10 mL fresh DI water and sonicated for 2 min to detach all bacteria adhering to the surfaces. Ten milliliters bacterial suspension was collected and spread onto an agar plate. The bacterial colonies were then quantified by culturing the bacteria on agar plates in the dark at 37 °C for 24 h. The films were then vortexed for 30 s in a 7:3:1 (v/v/v) mixture of isopropyl alcohol, chloroform, and formaldehyde (Duksan) to fix the bacteria onto the surfaces. The films were then immersed in acridine orange (Thermo Fisher Scientific) solution for 25 min and stained for fluorescence imaging. The bacteria adhering to the surfaces were detected by fluorescence microscopy (LSM 700; Carl Zeiss AG, Oberkochen, Germany).

The antimicrobial activity of basic amino acid-modified PI surfaces were evaluated using the LIVE/DEAD™ BAcLight™ Bacterial Viability Kit (Thermo Fisher Scientific). To confirm the bactericidal effect of the surfaces, bacterial suspensions (1 mL) were spread on each surface for 5 min then fixed with the aforementioned solution (7:3:1 (v/v/v) mixture of isopropyl alcohol, chloroform, and formaldehyde) without further washing. Live cells were stained green, and dead cells red in the LIVE/DEAD bacterial assay, subsequently confirmed by fluorescence microscopy (LSM 700; Carl Zeiss AG).

### Biofilm formation test

*E. coli* and *S. epidermidis* were cultivated for 12 h at 37 °C with 200 rpm stirring and transferred aliquots to the fresh Broth (20 g L^−1^ LB and 30 g L^−1^ TSB). Each specimen that was placed in the conical tube with bacteria transferred 20 mL fresh broth solution. Biofilm was grown on an incubator at 37 °C with static conditions for 48 h (*E. coli*) and 72 h (*S. epidermidis*). Biofilm formed specimens were washed 3 times with PBS solution and stained with acridine orange solution for 30 min. Biofilm characterization using confocal microscope with 80 μm stack scan mode (scanned at a step size of 1 μm). The fluoroscopic image was obtained using a laser wavelength of 488 nm (10 mW of illumination intensity) and cut off below wavelength of 500 nm to remove the backlight.

### Computational methods

The geometry of the molecules considered in this study was preliminarily optimized using the built-in Merck molecular force field (MMFF94) and Avogadro software [29]. The molecular electrostatic potential (MEP) was calculated using the Gaussian 09 program and density functional theory (DFT). The MEP of amino acids was calculated using the B3LYP/6-31g* basis set with geometry optimization and visualized using the GaussView 06 software [30].

### In vitro cytocompatibility

The cytotoxicity of each film to HaCaT and HDF cells was evaluated by direct and indirect methods according to the ISO 10993–5 standard (https://www.iso.org/standard/36406.html/). HaCaT and HDF cells were cultured in Dulbecco’s modified Eagle’s medium (DMEM; catalog no. SH30243, HyClone, Marlborough, Massachusetts, USA) with 10% (v/v) fetal bovine serum (FBS), 100 μg mL^−1^ penicillin, and 0.1 mg mL^−1^ streptomycin at 37 °C and 5% CO_2_ [31]. Before the cytotoxicity test, each film was sterilized via bilateral UV light exposure for 1 h.

Each film was then immersed in culture medium at 37 °C for 24 h and the extracted medium was passed through a 0.2 μm syringe filter. Cell viability was then evaluated by CCK-8 assay using Cell Counting kit-8 (CCK-8, Dojindo Laboratories, Kumamoto, Japan). Fresh medium was added to 48-well plates seeded at 2 × 10^5^ cells/well and incubated for 24 h.

A cell live/dead assay was performed using fluorescein diacetate (FDA)/propidium iodide fluorescence staining (Logos Biosystems, Anyang, Republic of Korea) to evaluate the viability of the cells attached to each film. HaCaT and HDF cells were seeded at a density of 2 × 10^5^ on each film (diameter = 12 mm) and cultured for 24 h. After several washed with PBS, the cells were fixed with 70% (v/v) cold ethanol and stained with 8 μg mL^−1^ FDA and 20 μg mL^−1^ propidium iodide. The stained HaCaT and HDF cells on the films were qualitatively examined by fluorescence microscopy (Axio Observer A1, Carl Zeiss AG, Jena, Germany).

### In vivo wound healing test

The wound healing effect of each film was determined for a 7-weeks CrljOri:CD1 (ICR) mouse wound defect model. The latter was established in accordance with the protocol of the Institutional Animal Care and Use Committee (No. PKNUIACUC-2021–29). The animals were housed at 40–70% relative humidity, 20–24 °C, and under a 12-h light–dark cycle [32].

The five treatment groups (control, PI, Leu, Lys, and Arg) were sterilized with an ultraviolet lamp before treatment as follows: Group 1: no treatment (control); Group 2: PI film treatment at wound site (PI group); Group 3: Leu film treatment at wound site (Leu group); Group 4: Lys film treatment at wound site (Lys group); and Group 5: Arg film treatment at wound site (Arg group).

All animal experiments were performed under isoflurane anesthesia. Each surgical site was disinfected with povidone-iodine and the skin wounds were made using a 5-mm biopsy punch. For all experimental groups, wound healing ability was confirmed by covering the wound with film after surgery and observing and imaging the wound size and condition with a digital camera at 0, 7, 10, and 21 days.

After 21 d, the animals were sacrificed and the wound tissues were collected, fixed in formalin, dehydrated, embedded in paraffin, sectioned at 5 μm thickness, mounted on slides, stained, rinsed, and dehydrated. The slides were stained with hematoxylin and eosin (H&E; H&E staining kit, Abcam, Cambridge, UK), Masson’s trichrome (MT; Masson's Trichrome stain kit, KTMRT, American MasterTech, Lodi, CA, USA), and Picrosirius Red (PSR; Sigma Aldrich) dyes. The stained slides were sealed with coverslips and MM24 mounting medium (Leica Biosystems, Wetzlar, Germany) and then examined under an optical microscope (LSM 700; Carl Zeiss AG, Oberkochen, Germany). Details of the staining methods are provided in the Supplementary Materials.

### Infectious wound healing test

The infectious wound healing efficacy of each film was evaluated using an ICR mouse model infected with *S. epidermidis*. To examine the reliability and effectiveness of each film in healing infected wounds, all groups were subjected to uniform skin defects with a biopsy punch (5 mm). In addition, wound infection was induced by applying 100 μL of *S. epidermidis* suspension (1 × 10^7^ CFU mL^−1^) to a defected skin area. Each film was covered and fixed with 3 M tegaderm, and the wounds were photographed on days 3, 7, 10 and 14 to observe the decrease in wound size.

The four treatment groups (PI, Leu, Lys, and Arg) were sterilized with an ultraviolet lamp before undergoing the following treatments: group 1, PI film treatment at wound site (PI group); group 2, Leu film treatment at wound site (Leu group); group 3, Lys film treatment at wound site (Lys group); and group 4, Arg film treatment at wound site (Arg group).

### Electrochemical measurements

The sensing performance and electrochemical characterization of the pH sensor were potentiometrically analyzed by electrochemical impedance spectroscopy (EIS; parstat 2273; Princeton Applied Research, Princeton, NJ, USA) in a two-electrode system. The analytical performance of the pH sensor was evaluated using open circuit potential and amplitude of 10 mV. The potential of the fabricated pH sensor was determined by immersing the device into a standard pH buffer solution (pH range 4–7) and washing it thoroughly with DI water.

The pH sensor workability test was conducted on fresh and infectious wounds under in vivo conditions using a 7-week-old ICR mouse. The infectious wound was established by continuously injecting the *S. epidermidis* suspension (3 × 10^8^ CFU mL^−1^) to confirm the workability of the pH sensor in wound conditions.

### In vivo stability test of amino acid-modified surfaces

In vivo stability of the amino acid bond on the surface was confirmed via a 1-week wound healing test. The stability of the amino acid-modified surfaces, which attached on the wound, was confirmed by detecting the amide bond of the amino acid-modified surface using ATR-FTIR spectroscopy at the beginning and after 7 days of attachment to the wound.

### Statistical analysis

All data were presented as mean ± standard deviation unless otherwise specified. Statistical significance was evaluated by means of the two-taled Student’s t-test between different groups. The level of significance was labeled as n.s.,*, **, and ***, representing non-significant and *p*-value of < 0.05, < 0.01, and < 0.001, respectively.

## Results

### Characterization of amino acid-modified surfaces

**Fig. 1:**
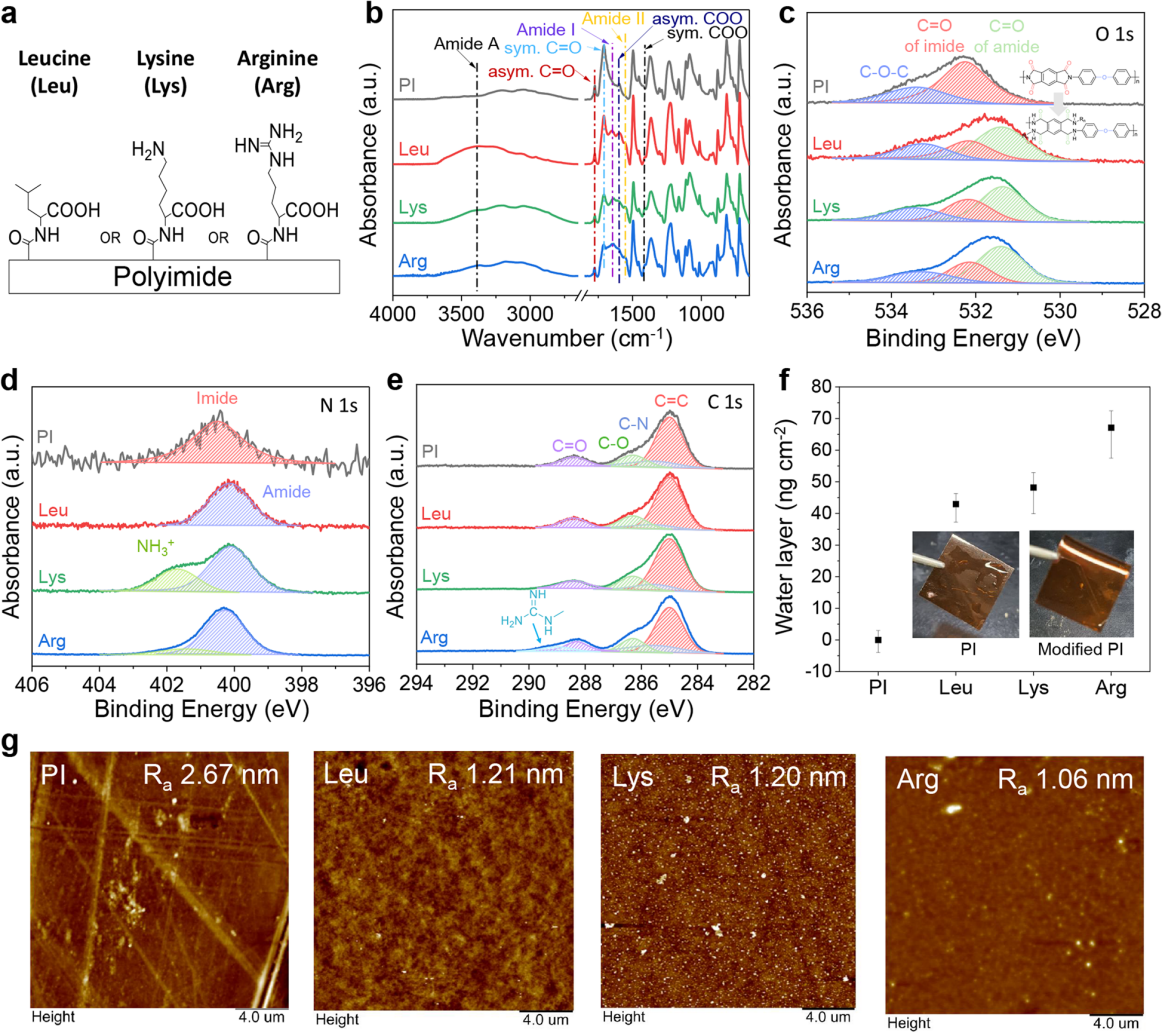
Surface characterization. (**a**) Schematic of amino acid-modified polyimide (PI) surfaces. (**b**) FT-IR spectra of amino acid-modified surfaces. High-resolution XPS of (**c**) O 1s peaks, (**d**) N 1s peaks, and (**e**) C 1s peaks of modified PI surfaces. (**f**) Water layer formation after amino acid modification. (**g**) Surface morphology of amino acid-modified PI surface determined by AFM measurement

Surface modifications with Lys, Arg (basic amino acids) and Leu (anionic amino acid for comparison) are shown in Fig. 1a. The main functional groups in amino acids-mediated surface modification were identified by attenuated total reflection Fourier transform infrared (ATR-FTIR) spectroscopy as described in Fig. 1b. PI without any further surface treatment or modification was designated PI. Symmetric and asymmetric C=O bonds of PI appeared at 1,710 cm^−1^ and 1,777 cm^−1^ and were significantly reduced by transformation to amide bonds via KOH treatment [26, 33]. The surface formed amide bonds with the amino acids, according to the peaks at 1,554 cm^−1^ (Amide I) and 1,651 cm^−1^ (Amide II) [26, 34]. Increases in the asymmetric and symmetric COO bonds of amino acids were observed at 1,590 cm^−1^ and 1,441 cm^−1^, respectively, while the aromatic ring of PI appeared at 1,597 cm^−1^ [26, 35, 36].

Details of the structural characteristics of amino acid-mediated modification were investigated by XPS (Fig. S3). In the high-resolution O 1s spectrum, an amide peak appeared as a result of binding between the carboxylic acids of PI and amino groups of the amino acids. Hence, the amino acids were grafted onto the surface through amide bonding (Fig. 1c) [26]. Figure 1d shows the High-resolution of N 1s spectrum with high NH_3_^+^ intensity at the Lys- modified surface, but not the Arg-modified surface [37] because of structural differences between Lys and Arg (Fig. S2). For the Arg-modified surface, the weak NH_3_^+^ intensity demonstrated that the amino acid was bonded mainly between the amino group bound at the alpha carbon and the carboxylic group at the PI surface, which is similar to protein-peptide structures [38]. The high-resolution C 1s spectrum showed a central carbon bonded with the three amino groups of Arg (Fig. 1e) [38]. Furthermore, the mass concentration of the N atom increased with the N atom ratio of the amino acids (Table S1).

A water absorption test was conducted and confirmed that the amino acids absorbed water and formed hydration layers which are important factors in the antifouling mechanism (Fig. 1f). The water absorption trend was consistent with the amphiphilic property of the amino acids (Table S2). Furthermore, Fig. 1g indicates that the amino acid-modified PI surface has a smooth surface.

### Surface potential characterization

**Fig. 2:**
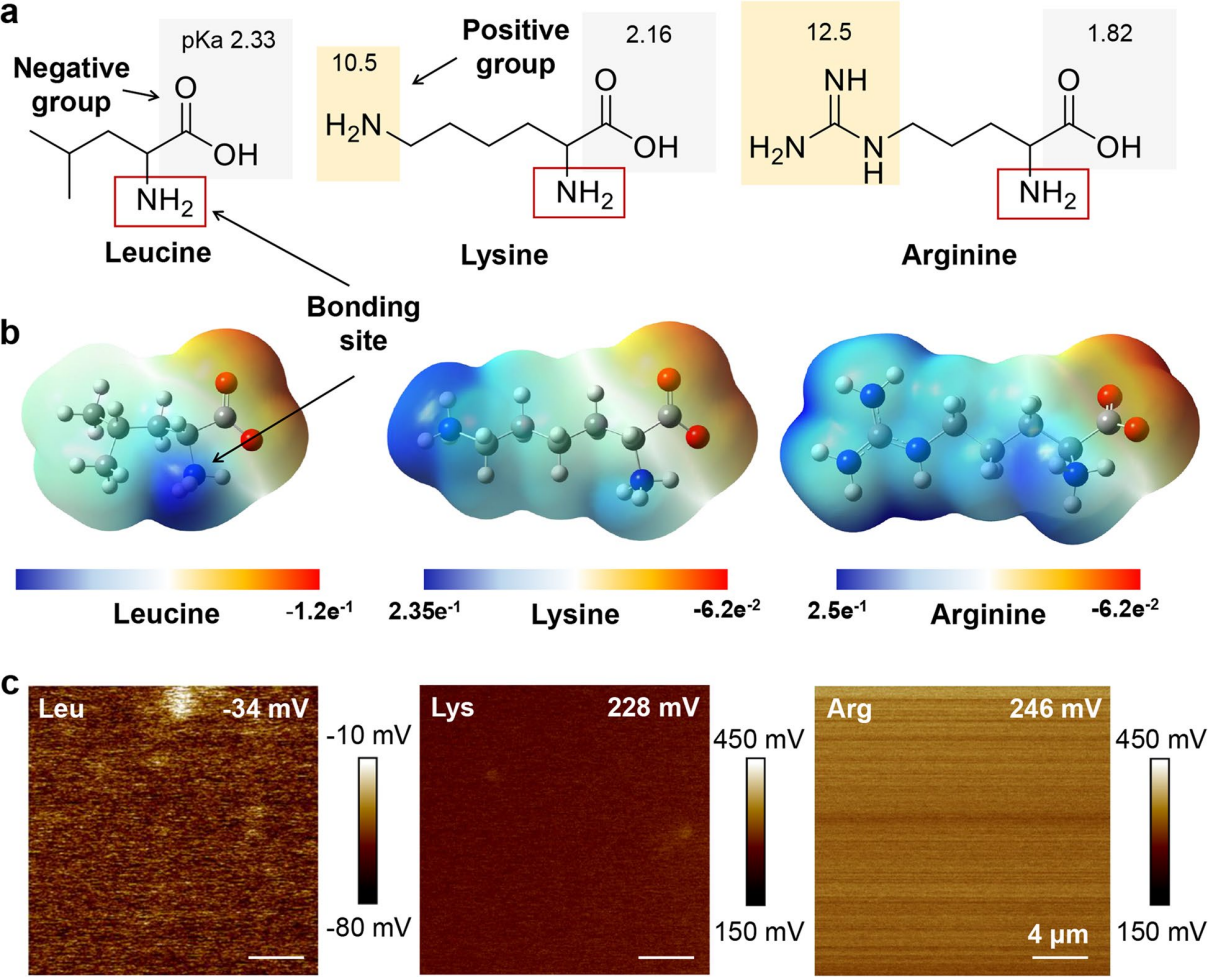
Surface potential characterization. (**a**) Chemical structures of amino acids. (**b**) Molecular electrostatic potential maps of amino acids. (**c**) Surface potentials of amino acid-modified surfaces measured by KPFM

The zwitterionic surface creates an electrostatic repulsion force which is vital to antifouling activity. Each amino group of basic amino acids has a different pKa, resulting in a different potential charge (Fig. 2a). Therefore, we validated the theoretical electrostatic potential of the functional group of amino acids by calculating the MEP via Gaussian 09 software (detailed DFT computational method depicted in methods) as shown in Fig. 2b. Leu has only a negative charge because of its methyl and carboxyl group, except for the amino group bound with the surface. Meanwhile, Lys and Arg have both negative and positive charges of functional groups, except for the amino group bound with the surface. Thus, Lys and Arg bounded on the surface can be expected to have zwitterionic characteristics.

The electrostatic potential of the surface based on the amino acids was measured by Kelvin probe force microscopy (KPFM), as shown in Figs. 2c and S4. Lue has only a single anionic group and -34 mV negative electrostatic potential in contrast with PI surface. However, Lys and Arg (basic amino acids) had 228 and 246 mV positive electrostatic potential, resulting in relatively positive zwitterionic surfaces. The electrostatic potentials of Lys and Arg vary with pH as they have intrinsically different structures (Fig. S5).

### Antimicrobial and biofilm inhibition test

**Fig. 3:**
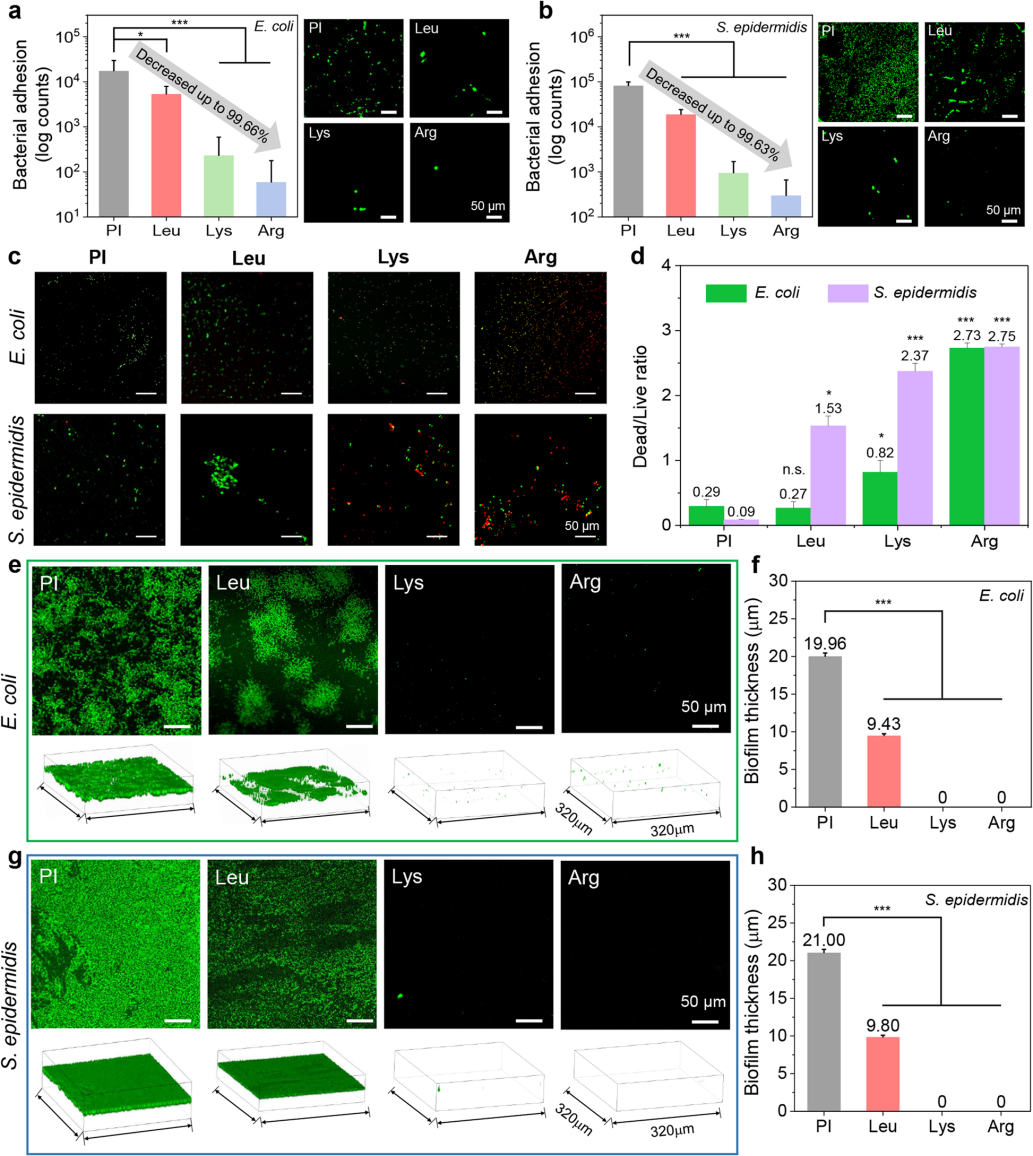
Antimicrobial and biofilm inhibition activity. Antifouling performance of amino acid-modified surfaces against (**a**) *E. coli* and (**b**) *S. epidermidis*. (**c**) Microscopic fluorescence images of live/dead bacteria assays. (**d**) Dead/live bacteria ratios. Biofilm inhibition test on (**e**) *E. coli* and (**g**) *S. epidermidis* (*p*-value in comparison to PI specimen). Biofilm thicknesses of (**f**) *E. coli* and (**h**) *S. epidermidis*

Bacterial adhesion is closely associated with surface biofilm formation on the surface and reliably reflects antibiofilm performance. Following that, we analyzed antimicrobial and biofilm inhibition of the amino acid-mediated modified surfaces (Fig. 3). Figure S6a shows the amount of *E. coli* attached to the surface revealed by exposure to the bacterial suspension. Compared to the PI, the basic amino acid-mediated modified surface showed a considerably reduced amount of surface-attached bacteria by up to 98.02%. Figure 3a shows the relative bacterial adhesion area (reduced by up to 99.66%, *p* < 0.001). Figure S6b shows the amount of bacteria attached to the specimen exposed to the *S. epidermidis* suspension. The modified surface reduced the amount of surface-attached bacteria by 97.09% (cf. Fig. S6a). The *S. epidermidis* adhesion area was reduced by up to 99.63%, indicating excellent antifouling properties (*p* < 0.001) (Fig. 3b). These results indicated that basic amino acid-modified surface suppressed bacterial adhesion to the surface and effectively inhibited adhesion and growth of both *E. coli* and *S. epidermidis*.

Wearable biomedical devices directly contact the human body. Their bactericidal activities are more important than prevention of bacterial adhesion as bacteria can grow in human sweat or body fluid. Here, the bactericidal activities of basic aminoacid-modified surfaces against *E. coli* and *S. epidermidis* were confirmed by live/dead assay (Figs. 3c and S7). The PI film and negatively charged Leu-modified surfaces showed abundant live but only sparse dead bacteria. Meanwhile, the Lys- and Arg-modified surfaces had substantial bacteria-killing activity because of their cationic amphiphilic characteristics. The dead/live ratios were calculated using the % of dead bacteria relative to the number of live bacteria (Fig. 3d). Arg obviously showed good bacterial killing performance against both *E. coli* (*p* < 0.001) and *S. epidermidis* (*p* < 0.001).

Figure 3e and g display *E. coli* and *S. epidermidis* biofilms and their 3D images, respectively. The fluorescence microscopic image shows significantly inhibited biofilm formation on basic amino acid-mediated modified surface (*E. coli*; *p* < 0.001, *S. epidermidis*; *p* < 0.001). The microscopic fluorescence images of biofilm formation were observed via ZEN 2011 software linked to a confocal microscopy (Fig. S8). Figure 3f shows the thickness of the *E. coli* biofilm on the surface of the PI and amino acid-modified surfaces. The thickness of the biofilm on the PI film, the Lue-, Lys-, and Arg-modified surfaces were 19.96, 9.43, 0 and 0 μm, respectively. And the thickness of the *S. epidermidis* biofilm on the PI film was formed on the PI film, the Leu-, Lys-, and Arg-modified surfaces were 21.00, 9.80, 0 and 0 μm, respectively (Fig. 3h).

### Biocompatibility & wound healing capacity test

**Fig. 4:**
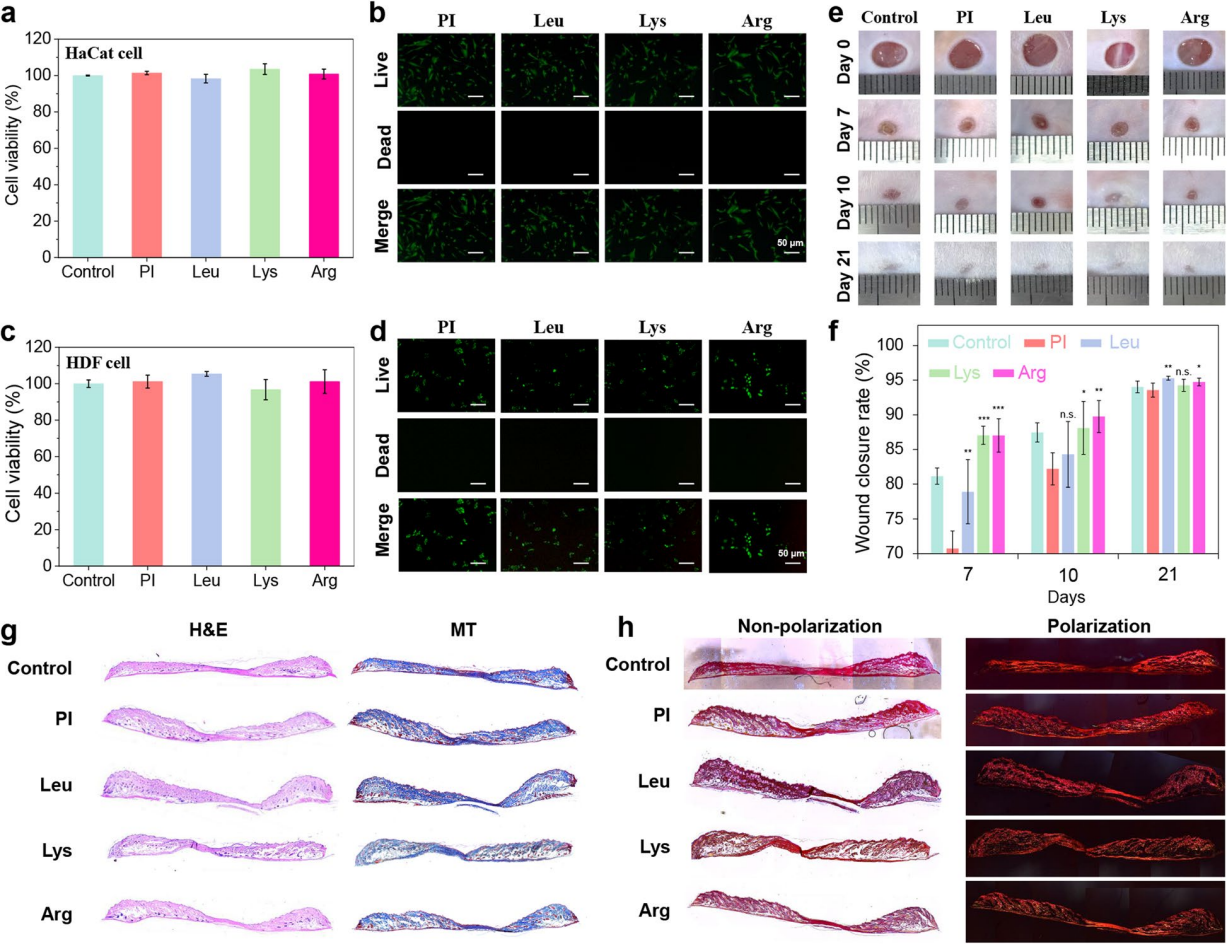
Biocompatibility and wound healing effect. (**a**) HaCat cell viability determined by indirect test. (**b**) Live/dead HaCat cell assay (direct test). (**c**) HDF cell viability determined by indirect test. (**d**) Live/dead HDF assay (direct test). (**e**) 21 days wound closure test. (**f**) Wound closure rates confirming wound healing effect (*p*-value in comparison to PI specimen). (**g**) H&E and MT staining of tissues subjected to amino acid-modified surfaces. (**h**) PSR staining of tissues subjected to amino acid-modified surfaces

We investigated indirect and direct cytotoxicity by CCK-8 assay and Cell Live/Dead assay, respectively. According to ISO 10993–5, a substance is indirectly cytotoxic if cell survival is < 70% that of the control. The indirect cytotoxicity tests (Fig. 4a and c) showed no significant difference in HaCaT and HDF cell viability compared with fresh medium after 1 day treatment. Direct cytotoxicity was evaluated by FDA and propidium iodide staining indicated live and dead cells, respectively. Observation of the stained cells under a fluorescence microscope revealed no dead cells in any specimen (Fig. 4b and d).

An ICR wound defect model was created to evaluate the effect of PI film modified with amino acid on wound healing progress at various time points. For the ICR model of PI, Lue, Lys, and Arg, wound healing on 0, 7, 10 and 21 days are shown in Fig. 4e. Measurements were made with Image J and are shown in Fig. 4f. Lys and Arg showed the significantly enhanced wound healing activities at 7 days (Lys; *p* < 0.001, Arg; *p* < 0.001) and 10 days (Lys; *p* < 0.05, Arg; *p* < 0.001) in comparison to the PI specimens. The reconstructed tissue was evaluated by H&E, MT, and PSR staining (Fig. 4g and h). Histological analyses of the control, PI, Leu, Lys, and Arg groups were performed through H&E, MT, and PSR staining of the recovered tissue on the 21st day after post-surgery. Complete healing was not achieved in all groups. Nevertheless, the group treated with the amino acid-modified PI film presented with relatively superior skin regeneration and hair follicle and sebaceous gland formation relative to the control and PI specimen. Under PSR staining, collagen type 1 appeared yellow/orange through a polarizing filter. The yellow/orange area was darker in amino acid-modified PI film group than the control and PI specimen. Thus, amino acid-modified PI promoted wound healing more effectively than the control and PI specimen.

### The workability test of the potentiometric pH sensor

**Fig. 5:**
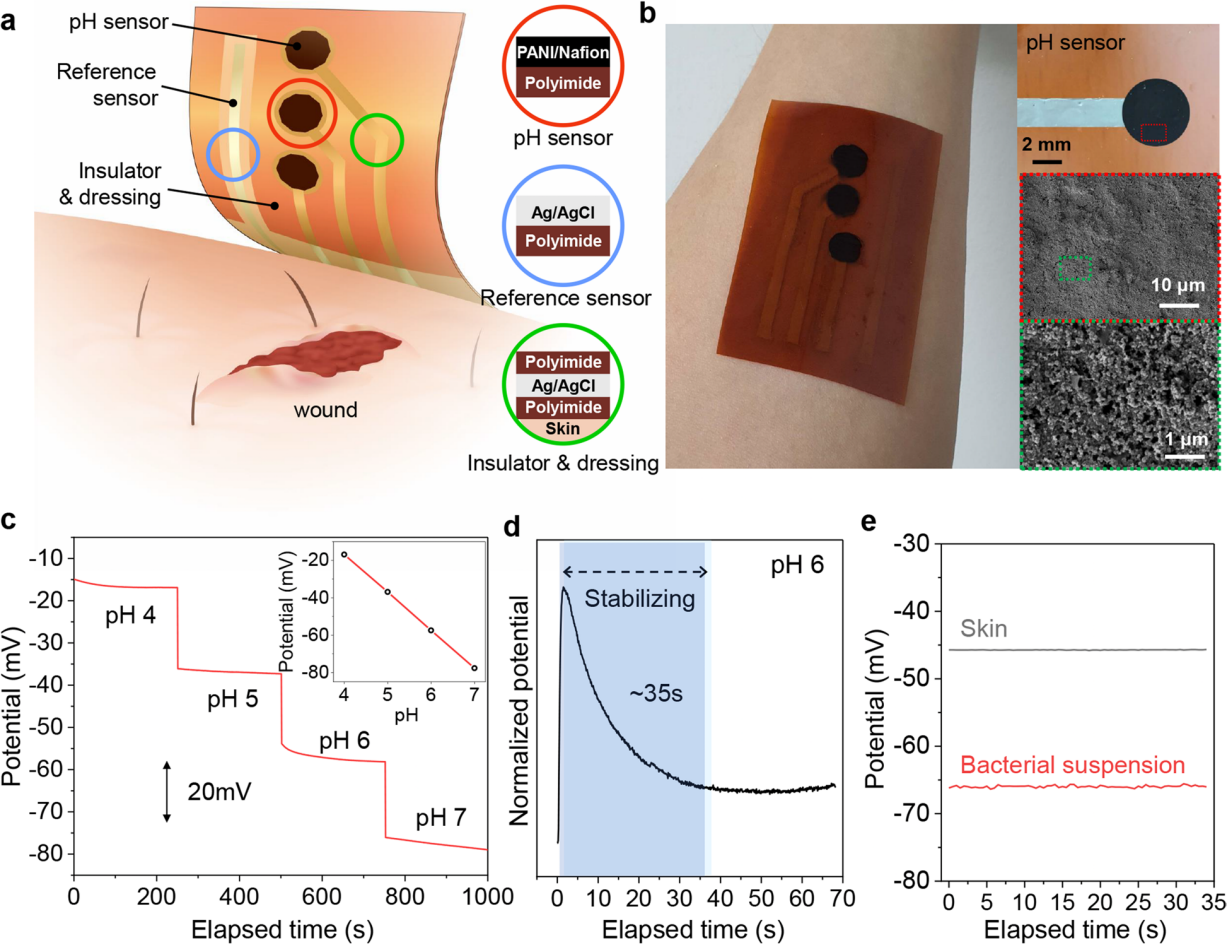
Workability of potentiometric pH sensor. (**a**) Schematic illustration of design of pH-monitorable wound healing devices to monitor wound status. (**b**) Image of pH sensor device on human skin. (**c**) Potentiometric analysis of pH sensors at various pH. (**d**) Stabilization time measurement of pH sensor. (**e**) Workability test of pH sensor on normal and bacterial suspension-contaminated human skin

A schematic representation of the potentiometric pH sensor developed on PI film basic with amino acids-mediated surface modification is shown in Fig. 5a. To monitor bacterial infections and promote wound healing, electrochemical approaches, including innate selectivity and sensitivity towards target analytes, were applied to transduce wound biomarkers concentration into potential. The pH sensor consisted of an Ag/AgCl reference electrode (Sigma Aldrich) and polyaniline (Sigma Aldrich) with Nafion resin (Sigma Aldrich) was deposited by screen printing. The pH sensor device is covered with a basic amino acid-modified PI for insulation and wound healing efficacy, except for the sensor electrode part.

A wearable pH sensor film attached to a human arm is illustrated in Fig. 5b. A layer of PANI/Nafion was deposited on a PI film (inset images). The electrode has a nanostructure that maximizes the electrochemically active surface area. The influence of the pH change on sensor detection is shown in Fig. 5c. The potentiometry of the pH sensor in buffer solution (pH 4, 5, 6, and 7) was measured. The response potential sensitivity was -20 mV pH^−1^. The time required for the electrode to stabilize was found to be less than 30 s (Fig. 5d). The skin pH was ~ 5.4 and the pH of the bacterial suspension deposited onto the skin was ~ 6.4 (Fig. 5e). The potentiometric measurements showed that pH sensor workability based on basic amino acid- modified PI film.

### Wound-healing pH sensor for monitoring the infectious wound-healing process

**Fig. 6:**
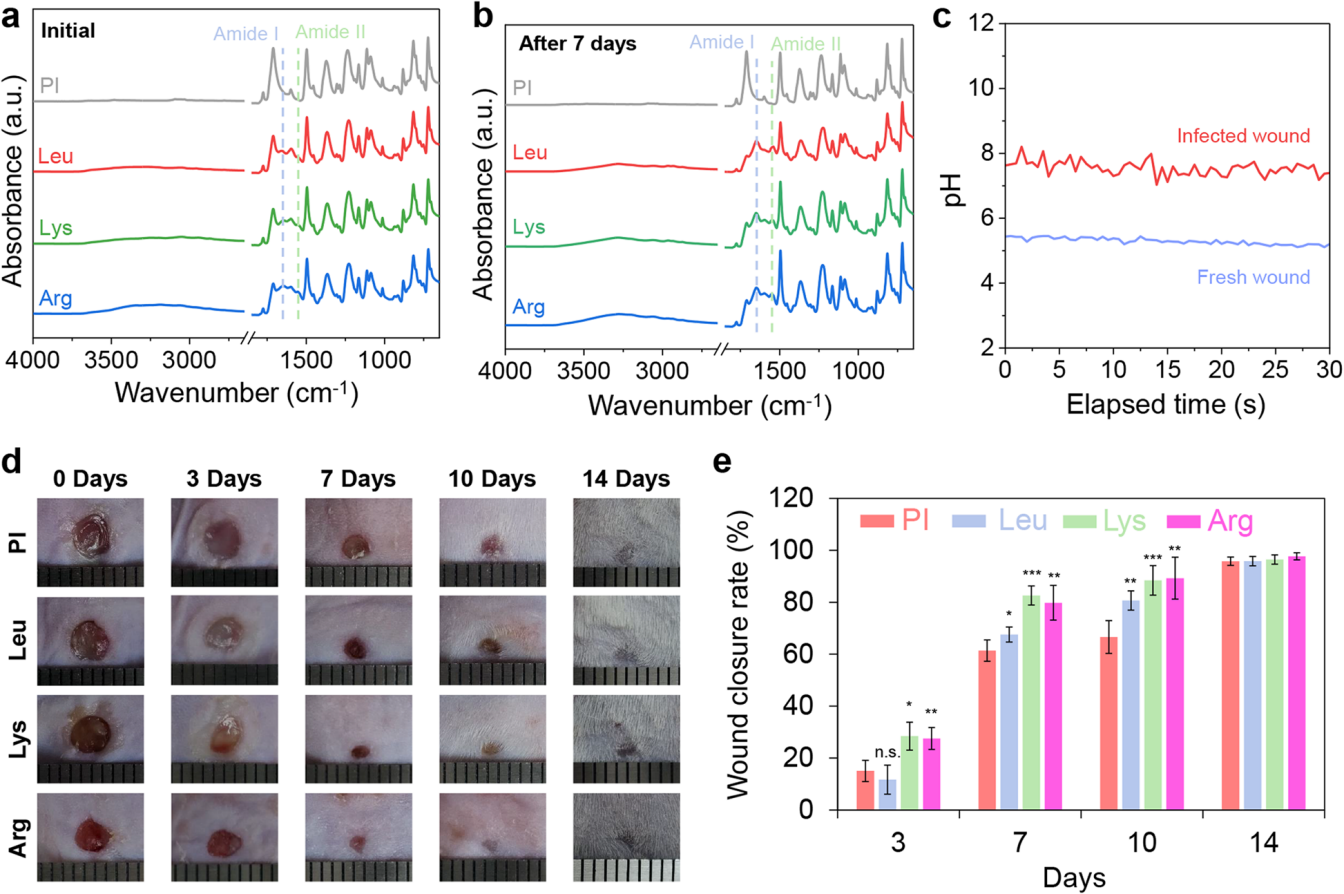
Application of wound healable pH sensor in vivo conditions. Stability of amino acid-modified surface during wound healing test at (**a**) the beginning and (**b**) after 7 days attachment to the wound. (**c**) Workability test of pH sensor under in vivo conditions. (**d**) Photographic images and (**e**) wound closure rate of infectious wound healing model during the wound healing test (*p*-value in comparison to PI specimen)

Figure 6a and b show the in vivo stability of amino acid bonds on the surfaces by detecting the amide bond using ATR-FTIR spectroscopy at the beginning and 7 days after the wound healing tests. After a 7-day wound healing test, it was confirmed that the amino acids were bound on the surface with the amide bonds unchanged (Fig. 6b). Figure 6c illustrates the workability of the pH sensor for monitoring the wound healing process of fresh and infectious wounds under in vivo conditions. The pH values of the fresh and infected wounds were approximately ~ 5.3 and ~ 7.5, respectively (Fig. 6c).

The wound healing efficacy of each film was investigated using an infectious wound healing model, and wound healing was almost complete after 14 days in all groups (Fig. 6d). Wound size reduction was determined as a percentage of wound area in each film treatment group. After 3, 7, 10, and 14 days of treatment, the wound recovery rates were 15.04, 61.39, 66.61, and 95.78% for the PI group, 11.68, 67.57, 80.66, and 95.83% for the Lys group, and 28.44, 82.62, 88.42, and 96.46% for the Lue group, and 27.52, 79.8, 89.28, and 97.7% for the Arg group, respectively (Fig. 6e). Briefly, the Lys and Arg treatment groups showed a promoted wound healing rate compared with the PI and Lue treatment groups owing to the inhibition of biofilm formation during the early stages of infection (at 7 days; *p* < 0.001, 10 days; *p* < 0.01 in compared with PI specimen).

## Discussion

Biomedical devices used in direct contact with skin or wounds, such as wound healing dressing, are important to manage the wound and monitor the status such as bacterial infection or condition deterioration during wound healing progress. Here, we used biocompatible basic amino acid-mediated PI surface modification to develop an antimicrobial wearable pH monitoring sensor with wound healing activity.

Basic amino acids such as Lys and Arg can be used in the surface zwitterionic functionalization of PI (Fig. 1). The basic amino acids-mediated modification process is biocompatible because it uses nontoxic amino acids and bonds on the PI surface with an amide bond. The reason is that biocompatible materials such as PEG could generate toxic substances by degradation in vivo conditions [39], whereas amino acids bound on PI to make an amide bond lead to biostable bonding in vivo conditions [40]. Based on the results of the present study, we established that the amino acid-mediated surface modification proceeded satisfactorily through a simple modification method with waterborne dipping method (Fig. 1b-e). The basic amino acid-mediated surface modification is an attractive method based on water-based modification without altering surface morphology (Fig. 1g) or intrinsic mechanical properties [26]. Furthermore, amino acid-mediate surface modification provided robust hygroscopic property by zwitterionic functional group. This zwitterionic surface could provide an antimicrobial effect and inhibit biofilm formation by hydration layer formation owing to the high hygroscopicity of zwitterions, which form an ~ 8 times stronger interaction with water molecules than that of hydrophilic polyethylene glycol [41]. Thus, basic amino acid-mediated zwitterionic functionalization should contribute to excellent antifouling performance by forming hydration layers.

The MEP calculations and KPFM measurements disclosed that basic amino acid-modified surfaces are zwitterionic surfaces and cationic amphiphilic surfaces (Fig. 2 and Table S2). This result suggested that Arg has relatively more suitable for antimicrobial surfaces in terms of bactericidal cationic amphiphilic surfaces [42, 43] because cationic amphiphilic surfaces interact electrostatically with the negatively charged bacterial phospholipids, destroy cell walls and cause cell contents to leak and cells to die by a mechanism similar to that of cationic antimicrobial peptides [39, 44]. Therefore, the results of this study indicated that basic amino acid-mediated surface modification has good antimicrobial and biofilm inhibition performances, forms hydration layer formation by hydrophilicity, generates electrostatic repulsion via zwitterionic characteristics and has bactericidal efficacy via its positively charged surface with cationic amphiphilic properties.

The antimicrobial test against *E. coli* and *S. epidermidis* indicated that basic amino acid-modified PI surfaces have excellent antimicrobial, antifouling and bactericidal performance (Fig. 3a-d and S6). These results showed the ability to prevent infection of basic amino acid-modified PI surfaces from bacterial contamination. Moreover, the biofilm inhibition test against *E. coli* and *S. epidermidis* showed more reliable infection inhibition capability in Fig. 3e and f. The infections associated with biofilm formation at healthcare devices could induce a long period of hospitalization. In this study, Arg, in particular, had good bactericidal activity against both *E. coli* and *S. epidermidis*, which rendered its applicability in antimicrobial healthcare devices. Furthermore, antimicrobial and biofilm inhibition performances make the basic amino acid-modified surfaces of pH sensor could be applicable for actual wound treatment in human skin with safety via preventing infection from bacterial contamination and biofilm formation.

Cytocompatibility is a critical characteristic of biomedical devices that come into contact with wounds. The cell cytotoxicity tests on HDF (human dermal fibroblasts) and HaCat (human epithelial keratinocytes) confirmed that demonstrated that basic amino acid-modified surfaces are biocompatible in pH-monitorable wound healing dressings that enter into direct contact with skin wounds (Fig. 4a-d). Furthermore, the wound healing test and tissue staining results revealed the superior wound healing efficacy of basic amino acid-modified pH-monitorable wound healing dressing film compared with that of PI film (Figs. 4e-h). Following these results, PI film modified with basic amino acids was nontoxic to HaCaT, and HDF supported promising candidate materials for pH-monitorable wound healing dressing with biocompatibility.

The pH within wound conditions is a significant biochemical signal and an important factor in wound healing progress. Normal skin and wounds have a pH in the range of 4–6, however, the pH of the infected wounds becomes alkaline > 6.5 primarily in response to the presence of bacteria. Thus, pH is an essential diagnostic parameter for infection. The workability test of pH-monitorable wound healing dressing based on basic amino acid-modified PI films was conducted in the 4–7 range. The workability of the fabricated pH sensor, which was created on basic amino acid-modified PI film, was confirmed by measuring the electrochemical potential for pH measurement and its stabilization time (Fig. 5c and d). Furthermore, the potential measurements of bacterial suspension and normal human skin were validated to be applicable for monitoring wound infection (Fig. 5e). These results suggested that pH-monitorable wound healing dressings could be applicable for monitoring human skin potentially. The discoveries of the present study could be expanded to various wearable healthcare devices designed to monitor and promote healing and prevent infection in wounds.

The stability of amino acid-modified surfaces during the wound healing process could secure the usability and reliability of pH-monitorable wound healing dressings for use in wound management. Figure 6a and b show the amide bonds to demonstrate the adequate durability of the bonds between the amino acid and the surface during the wound healing process. As shown in the ATR-FTIR spectra, the amide bond of the amino acid-modified PI film was still detectable after 7 days of conducting the wound healing test (Fig. 6b), compared to the initial condition (Fig. 6a). This result demonstrates that the amino acid-modified surface could be applied for a relatively longer duration than that of conventional wound healing dressings (< 5 days) [14].

To evaluate their practical applicability in wound management, the wound healing effect was evaluated using an infectious wound healing model. The infectious wound healing model could represent the potential of using pH-monitorable wound healing dressings in real-world healthcare devices. As shown in Fig. 6c, the fabricated pH-monitorable wound healing dressing demonstrated good workability in fresh and infected wounds. The pH values of the fresh (~ 5.3) and infected wounds (~ 7.5) confirmed workability under in vivo conditions, which coincided with previously reported findings [45].

Furthermore, an infectious wound healing test was performed to evaluate the synergistic effect of the antibacterial and wound healing properties of basic amino acid-modified surfaces (Fig. 6d and e). Owing to their lack of bactericidal effects, the PI and Leu films showed reduced effects on wound closure during the infectious wound healing test compared with the normal wound healing test (PI, decreased from 70.72% to 61.39%; Leu; decreased from 78.92% to 67.57% on day 7), as shown in Fig. 4f. Meanwhile, it is important to note that Lys and Arg retained their wound healing effects in the infectious wound healing model owing to the bactericidal effect of the cationic amphiphilic surfaces, as demonstrated in Fig. 3. Thus, basic amino acid-mediated cationic amphiphilic surfaces can be used for the safe management of wounds with prevention of bacterial infection and may serve as a viable and effective approach for enhancing the techniques of wound care as well as wearable biomedical devices.

## Conclusions

In summary, the pH-monitorable wound healing dressing based on the basic amino acid-modified PI surfaces showed excellent antimicrobial, antibiofilm, and wound healing efficacy and effectively monitored wound infection. The basic amino acids with Lys and Arg could be provided cationic amphiphilic surface that contribute zwitterionic functional groups on the PI surface. Hence, they facilitate bacteria killing and have antibiofilm activity similar to that of cationic antimicrobial peptides. Lys- and Arg-mediated PI surfaces had high wound healing efficacy, biocompatibility, hydrophilicity, and could prevent infection. Furthermore, pH sensor fabricated in the form of basic amino acid-modified PI surfaces work well under various pH conditions and bacterial infection levels on human skin. The findings of this study are expected to contribute to the fields of wound management and wearable healthcare devices. However, this work is focused on the role of surface modification based on basic amino acids; thus, further research regarding the combination with wound healing promoters is required to allow for practical use of real wound healing devices with pH-monitoring function.

## Supplementary Information

**Additional file 1.**

## Data Availability

The datasets and materials used and/or analyzed during the current study are available from the corresponding author on reasonable request.

## Abbreviations

XPS: X-ray photoelectron spectroscopy
KPFM: Kelvin probe force microscopy
AFM: Atomic force microscopy
Lys: Lysine
Arg: Arginine
Leu: Leucine
*E. coli*: *Escherichia coli*
*S. epidermidis*: *Staphyloccocus* *epidermidis*
PI: Polyimide
DI: Water Distilled water
MES: 2-(N-morpholino)ethanesulfonic acid
EDC: 1-ethyl-3-(3-dimethylaminopropyl)carbodiimide
NHS: N-hydroxysuccinimide
ATR-FTIR: Attenuated total reflection-Fourier transform infrared
LB: Luria Bertani
TSB: Tryptic soy broth
DFT: Density functional theory
MEP: Molecular electrostatic potential
EIS: Electrochemical impedance spectroscopy
FDA: Fluorescien diacetate
H&E: Hematoxylin and Eosin
PSR: Picrosirius red
MT: Masson’s trichrome
