# Supplementary material for: Basic amino acid-mediated cationic amphiphilic surfaces for antimicrobial pH monitoring sensor with wound healing effects
Source: Biomater Res. 2023 Feb 17;27:14. doi: 10.1186/s40824-023-00355-0 (PMC9936651; doi:10.1186/s40824-023-00355-0)
Supplement: Supplementary file 1 — Additional file 1. [file 40824_2023_355_MOESM1_ESM.pdf]

Supplementary Materials

Basic amino acid-mediated cationic amphiphilic surfaces for antimicrobial pH monitoring sensor with wound healing effects

Dong Uk Lee[a,b], Se-Chang Kim[c,d], Dong Yun Choi[a,*], Won-Kyo Jung[c,d,e,*], Myung Jun Moon[b,*]

[a]Biomedical Manufacturing Technology Center, Korea Institute of Industrial Technology, Yeongcheon, 38822, Republic of Korea

[b]Department of Industrial Chemistry, Pukyong National University, Busan 48513, Republic of Korea

Institute of Industrial Technology, Yeongcheon 38822, Republic of Korea

[c]Department of Biomedical Engineering, and New-senior Healthcare Innovation Center (BK21 Plus) Pukyong National University, Busan 48513, Republic of Korea

[d]Research Center for Marine Integrated Bionics Technology, Pukyong National University, Busan 48513, Republic of Korea

[5]Research Center for Marine Integrated Bionics Technology, Pukyong National University, Busan 48513, Republic of Korea

D. U. Lee and S. Kim contributed equally to this work.

Corresponding author. Email: dychoi311@kitech.re.kr (D. Y. Choi), wkjung@pknu.ac.kr (W. Jung), mjmoon@pknu.ac.kr (M. J. Moon)

Materials and Method

Histomorphological evaluation

Hematoxylin and eosin (H&E) staining

Animals were sacrificed on day 21 after surgery and their wounds and surrounding tissues were collected, fixed in formalin, processed, embedded, sectioned to 5 μm thickness with a microtome (HistoCore MULTICUT, Leica Microsystems UK Ltd., Milton Keynes, UK), mounted on slides, dewaxed and rehydrated, treated with hematoxylin and eosin for nuclear and cytoplasm staining, dehydrated with xylene, sealed with cover slips and MM24 mounting medium (Leica Biosystems, Wetzlar, Germany), and observed under an optical microscope (Leica Microsystems UK Ltd., Milton Keynes, UK).

Masson's trichrome (MT) staining

The slides were subjected to Masson's trichrome staining (KTMRT; American MasterTech, Lodi, CA, USA) to examine connective tissue and collagen. Briefly, dewaxed tissue sections were immersed in preheated (60 °C) Bouin's fluid for 60 min, Weigert's hematoxylin for 5 min, Biebrich scarlet for 15 min, tungsten phosphorus/molybdic acid phosphorus for 10 min, aniline blue solution for 5 min, and 1% (v/v) acetic acid for 1 min, washed, dehydrated with xylene, sealed with cover slips and MM24 mounting medium, and observed under an optical microscope (Leica Microsystems UK Ltd., Milton Keynes, UK).

Picrosirius Red (PSR) staining

The slides were stained with Picrosirius Red (PSR) to evaluate collagen deposition at the wound sites. They were hydrated, dewaxed, immersed in hematoxylin for 8 min, immersed in PSR solution for 60 min, dehydrated with xylene, sealed with cover slips and MM24 mounting medium, and observed under an optical microscope fitted with a polarizing filter.

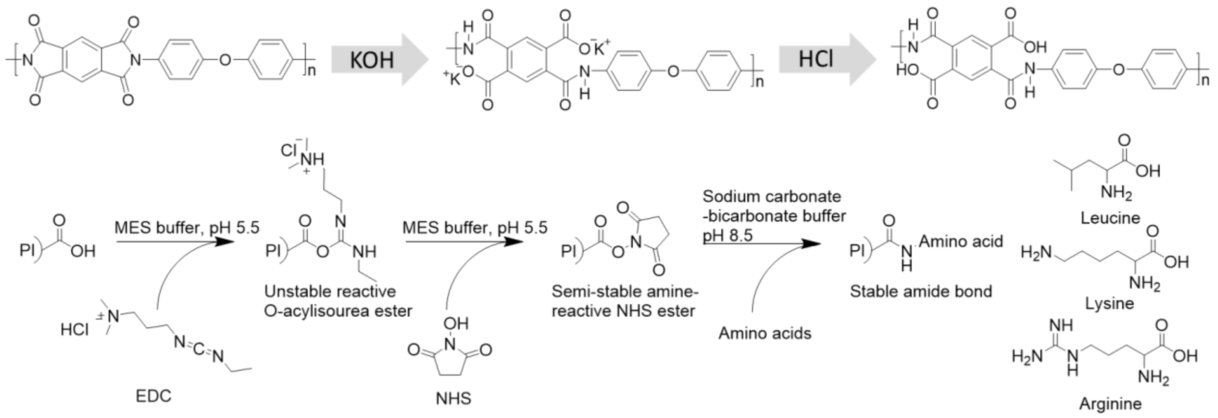

Fig. S1. Schematic illustration of polyimide (PI) surface modification via EDC/NHS peptide coupling reaction.

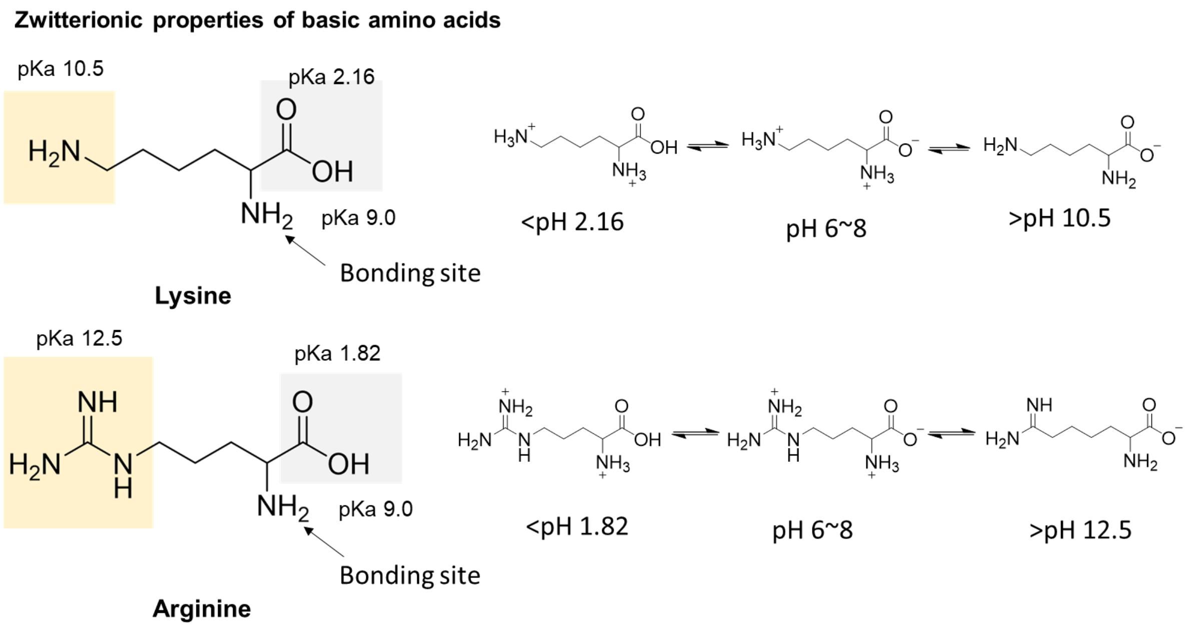

Fig. S2. Chemical structures of pH dependent basic amino acids.

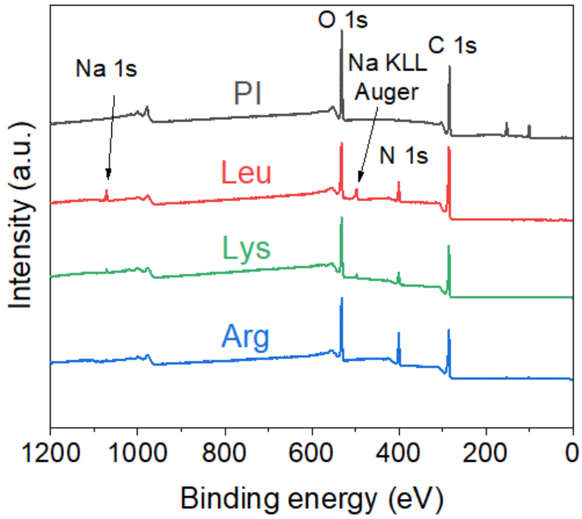

Fig. S3. XPS spectra of various polyimide surfaces modified with Leu, Lys, and Arg.

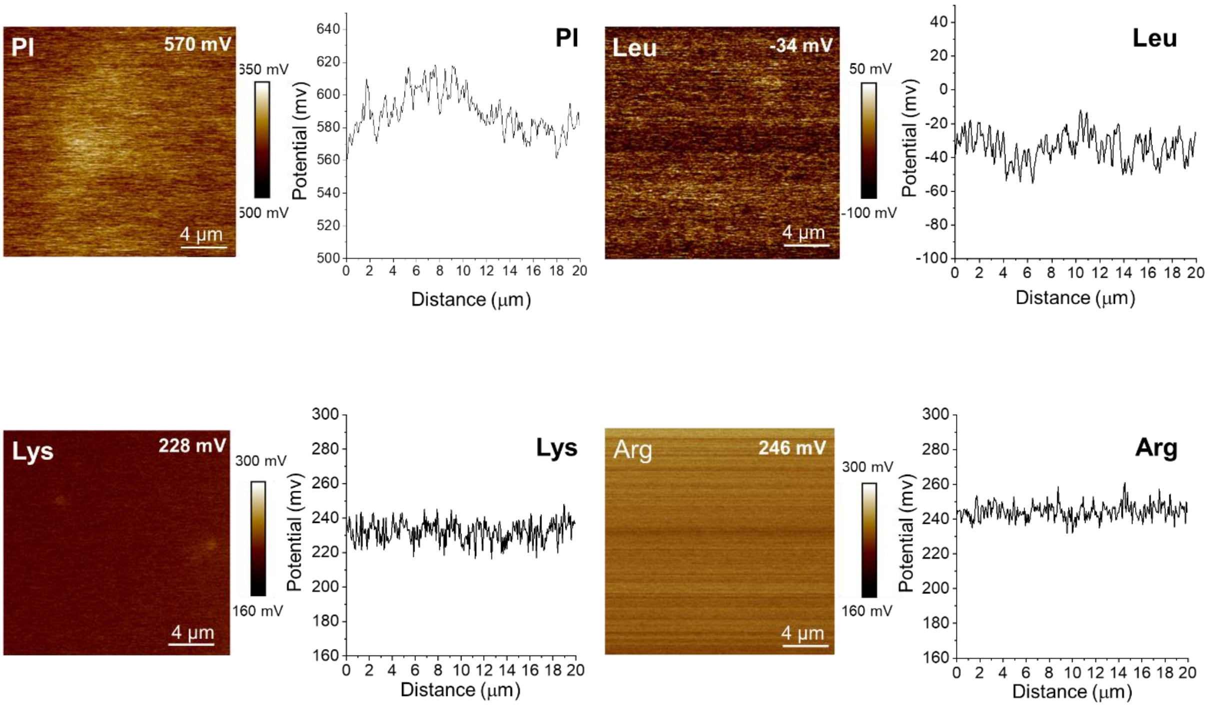

Fig. S4. Surface potentials of various modified PI surfaces determined by KPFM measurement.

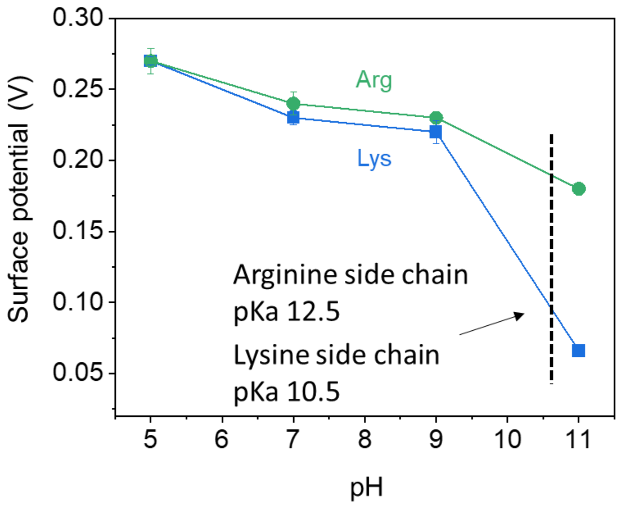

Fig. S5. Surface potentials of Arg- and Lys-modified PI surfaces at various pH.

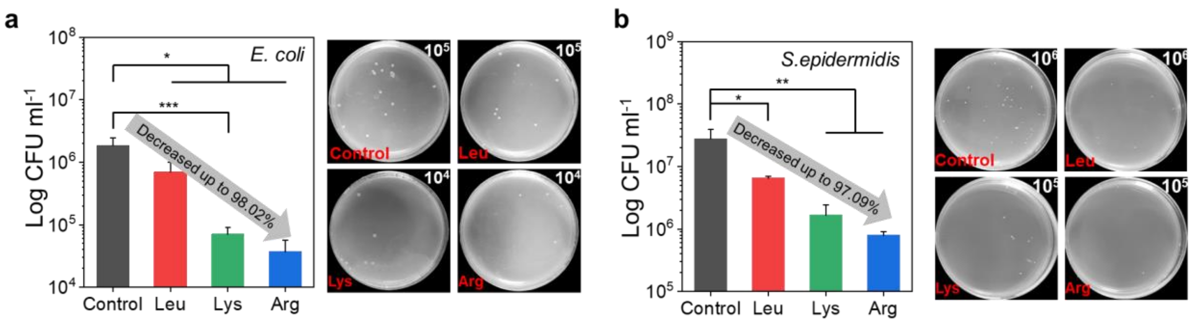

Fig. S6. Agar plate test quantification of *E. coli* and *S. epidermidis* adhesion to various surfaces.

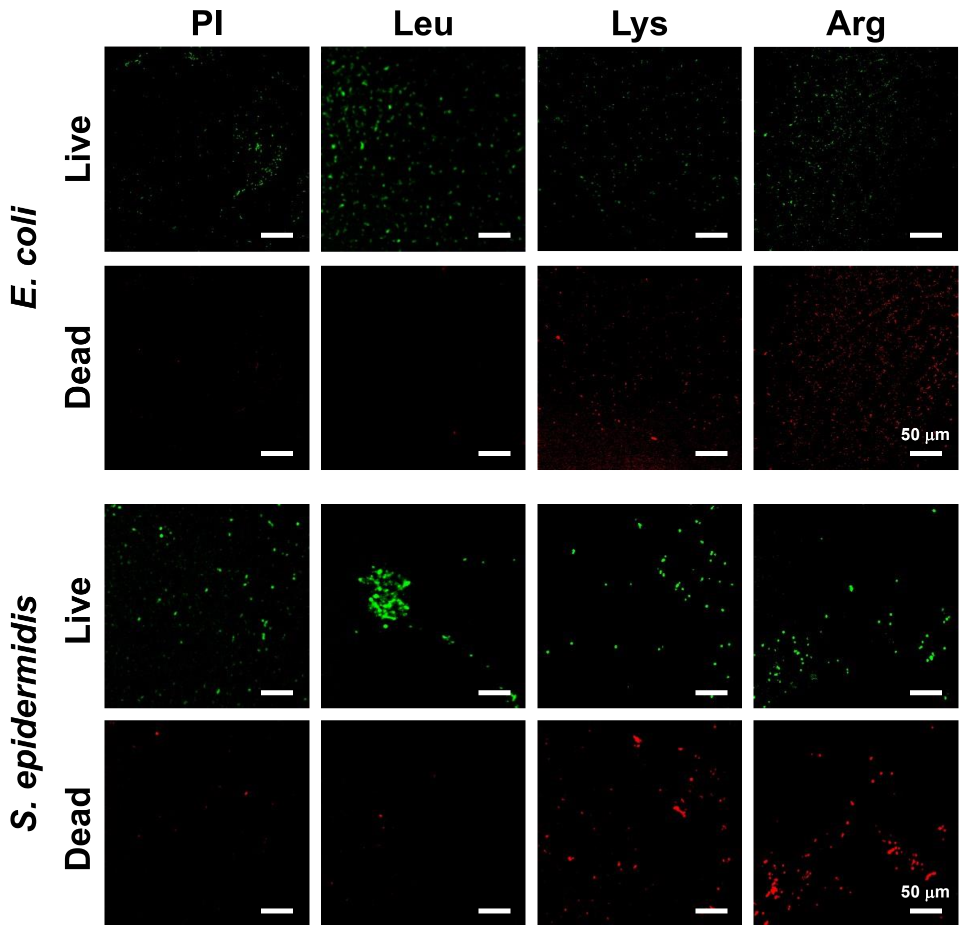

Fig. S7. Microscopic fluorescence images of surface bactericidal efficiency determined by live/dead assay.

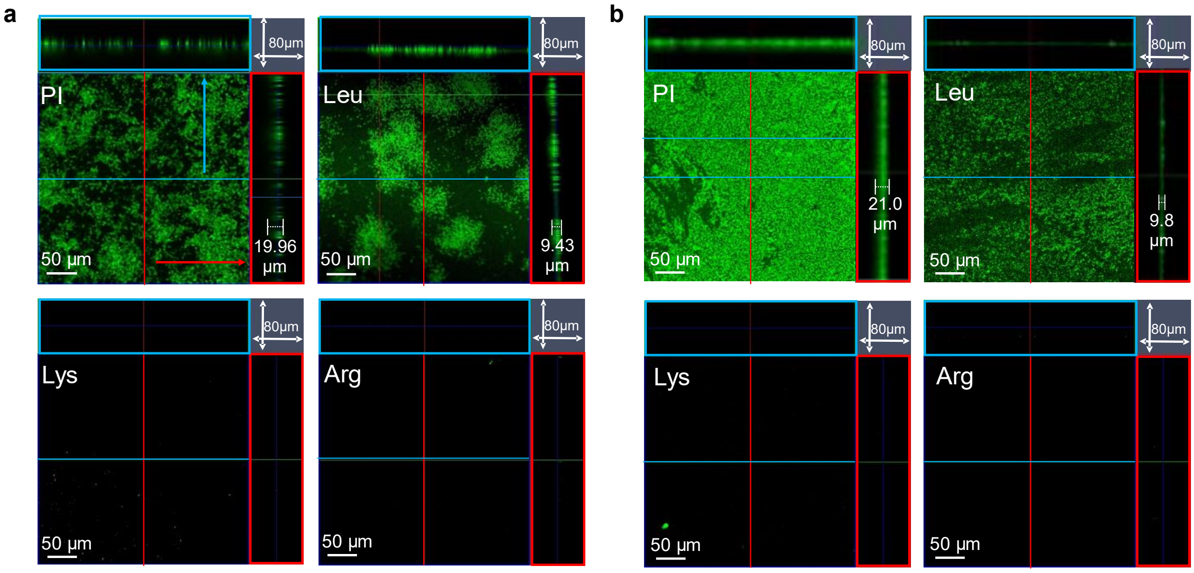

Fig. S8. Orthogonal confocal microscopic images of biofilm inhibition test.

Table S1. Surface compositions of amino acid-modified PI surfaces.

	C	N	O	Note.
PI	56.76*	1.26	41.98	Reference
Leu	68.74	8.78	22.48	$C_6H_{13}NO_2$
Lys	58.40	11.0	30.7	$C_6H_{14}N_2O_2$
Arg	44.80	18.40	36.8	$C_6H_{14}N_4O_2$

*Mass concentration %

XPS composition showed that the N atom is increased following the intrinsic N atom ratio of amino acids.

Table S2. Surface energy values determined by contact angle measurement in different solvents.

Specimen	Contact angle, °		Surface energy, mN/m		
	water	glycerol	γ_S	γ_S^d	γ_S^p
PI	53.37	41.25	45.88	7.72	38.16
Leu	37.42	38.68	68.76	2.39	66.37
Lys	26.0	35.38	81.00	1.32	79.68
Arg	10.29	19.06	85.17	2.41	82.76

*Surface energy was calculated by Owens Wendt Rabel and Kaeble model with water and glycerol

The contact angle was measured using a contact angle analyzer (Phoenix 150, SEO, Korea) at 20 °C and 40% relative humidity, and a water droplet (14 µL) was injected into the polyimide surfaces. The changes in the water contact angle were noted at different time points. Surface energy was calculated by the Owens, Wendt, Rabel, and Kaeble models using the contact angles of DI water and glycerol, as shown in below equation 1.

$$\frac{\gamma_L(1+cos\theta)}{2\sqrt{\gamma_L^d}} = \sqrt{\gamma_S^p}\cdot\sqrt{\frac{\gamma_L^p}{\gamma_L^d}} + \sqrt{\gamma_S^d} \qquad (1)$$

where θ is the contact angle, γ_L is the surface tension of the liquid, γ_s is the surface free energy of the solid, γ^d is the dispersive component of the surface energy, and γ^p is the polar component of surface energy.

To compare the water layer formation on the surface according to the hydrophilicity of the surface, each polyimide film (10 × 10 cm) was immersed in DI water for 5 min; the moisture on the surface was wiped off well and then the weight was measured. This procedure was performed at least 5 times for all specimens and compared. [1]

Reference

1. Lee DU, Kim DW, Lee SY, Choi DY, Choi SY, Moon KS, et al. Amino acid-mediated negatively charged surface improve antifouling and tribological characteristics for medical applications. Colloids Surf B Biointerfaces [Internet]. Elsevier B.V.; 2022;211:112314. Available from: https://doi.org/10.1016/j.colsurfb.2021.112314
